# Neural plasticity in schizophrenia: An integrated approach for rehabilitation by means of tms and cognitive remediation training

**DOI:** 10.1192/j.eurpsy.2023.577

**Published:** 2023-07-19

**Authors:** S. Torriero, C. Gesi, A. Vergallito, C. Mencacci, B. Dell’Osso

**Affiliations:** ^1^ ASST Fatebenefratelli Sacco; ^2^Department of Psychology, University of Milan Bicocca; ^3^Department of Biomedical and Clinical Sciences Luigi Sacco; ^4^“Aldo Ravelli” Center for Neurotechnology and Brain Therapeutic, University of Milan, Milan, Italy; ^5^Department of Psychiatry and Behavioural Sciences, Stanford University, Stanford, United States

## Abstract

**Introduction:**

Schizophrenia is a severe and disabling psychiatric disorder probably based on complex pathophysiological mechanisms of reduced inhibition, impaired connectivity and reduced plasticity in neural networks. Beside clinical symptomatology, a core feature of schizophrenia is a global cognitive and social disability, which strongly affect patients’ lives and their quality of life. The cognitive impairment involves memory, attention, executive functions, language, facial emotion recognition and theory of mind abilities. Cognitive remediation strategies, in addition to pharmacological and psychological treatments, has received increasing attention in recent years, as well as the use of non-invasive brain stimulation techniques such as TMS, which have demonstrated promising therapeutic potential.

**Objectives:**

The present study aimed to evaluate the efficacy of TMS to induce improvements in cognitive functioning in schizophrenia. It also aimed to test the effects of a combined approach to rehabilitation, using both TMS and cognitive remediation strategies.

**Methods:**

16 patients were submitted to effective or sham iTBS over the left dorsolateral prefrontal cortex during 3 consecutive weeks. In half of patients the neuromodulation was combined with daily cognitive remediation training (Cogpack software), administered immediately after the application of TMS. Clinical, cognitive and social functioning were tested at baseline and at different timepoints after conclusion of the rehabilitation protocol (immediately after the 3 weeks protocol, and after 1, 3 and 6 months).

**Results:**

The preliminary results indicate that the proposed TMS protocol induced significant improvements in global cognition. In addition, patients submitted to TMS, even without combined cognitive rehabilitation training, showed major benefits after 1 month from brain stimulation.

**Conclusions:**

These preliminary data suggest that TMS can induce long-lasting plastic changes in the prefrontal cortex of schizophrenic patients, improving their cognitive perfomances. TMS could be therefore considered in the treatment of schizophrenia to reduce cognitive impairments.

**Disclosure of Interest:**

None Declared

